# A prospective, non-randomized, no placebo-controlled, phase Ib clinical trial to study the safety of the adipose derived stromal cells-stromal vascular fraction in idiopathic pulmonary fibrosis

**DOI:** 10.1186/1479-5876-11-171

**Published:** 2013-07-15

**Authors:** Argyris Tzouvelekis, Vassilis Paspaliaris, George Koliakos, Paschalis Ntolios, Evangelos Bouros, Anastasia Oikonomou, Athanassios Zissimopoulos, Nikolaos Boussios, Brian Dardzinski, Dimitrios Gritzalis, Antonis Antoniadis, Marios Froudarakis, George Kolios, Demosthenes Bouros

**Affiliations:** 1 Department of Pneumonology, Medical School, Democritus University of Thrace, Alexandroupolis, Greece; 2 Adistem Ltd., Wanchai, Hong Kong; 3 Hellenic National research foundation stem cell bank, Athens, Greece; 4 Biohellenika SA, Thessaloniki, Greece; 5 Laboratory of Pharmacology, Medical School, Democritus University of Thrace, Alexandroupolis, Greece; 6 Department of Radiology, Medical School, Democritus University of Thrace, Alexandroupolis, Greece; 7 Department of Nuclear Medicine, Medical School, Democritus University of Thrace, Alexandroupolis, Greece; 8 Iaso General Hospital, Athens, Greece; 9 Department of Pneumonology, General Hospital of Serres, Serres, Greece; 10 Department of Pneumonology, University Hospital of Alexandroupolis, Democritus University of Thrace, Alexandroupolis 68100, Greece

**Keywords:** Adipose derived stromal cells, Mesenchymal stem cells, Idiopathic pulmonary fibrosis, Safety, Clinical trial, Stromal vascular fraction

## Abstract

**Introduction:**

Regenerative medicine and particular adult stem cells represent an alternative option with several fruitful therapeutic applications in patients suffering from chronic lung diseases including idiopathic pulmonary fibrosis (IPF). Nevertheless, lack of knowledge regarding the origin and the potential of mesenchymal stem cells (MSCs) to differentiate into fibroblasts has limited their use for the treatment of this dismal disease.

**Patients and methods:**

To this end, we conducted a phase Ib, non-randomized, clinical trial to study the safety of three endobronchial infusions of autologous adipose derived stromal cells (ADSCs)-stromal vascular fraction (SVF) (0.5 million cells per kgr of body weight per infusion) in patients with IPF (n=14) of mild to moderate disease severity (forced vital capacity –FVC>50% predicted value and diffusion lung capacity for carbon monoxide-DL_CO_>35% of predicted value). Our primary end-point was incidence of treatment emergent adverse events within 12 months. Alterations of functional, exercise capacity and quality of life parameters at serial time points (baseline, 6 and 12 months after first infusion) were exploratory secondary end-points.

**Results:**

No cases of serious or clinically meaningful adverse events including short-term infusional toxicities as well as long-term ectopic tissue formation were recorded in all patients. Detailed safety monitoring through several time-points indicated that cell-treated patients did not deteriorate in both functional parameters and indicators of quality of life.

**Conclusions:**

The clinical trial met its primary objective demonstrating an acceptable safety profile of endobronchially administered autologous ADSCs-SVF. Our findings accelerate the rapidly expanded scientific knowledge and indicate a way towards future efficacy trials.

## Introduction

Idiopathic pulmonary fibrosis (IPF) is a devastating, fibroproliferative chronic lung disorder with complex and yet unknown disease biology. As a result, there is no current standard of care for patients with IPF since both the disease and the effort to treat it are moving targets [1,2]. The specific pathogenetic pathway, type of cells or cellular products that should be targeted are under debate. This lack of information has led physicians to apply a more oncologic approach with the administration of drug regimens that inhibit multiple pathogenetic pathways, including pirfenidone [3,4] and tyrosine kinase inhibitors [5], with potential important side-effects. Despite extensive research efforts and large multicenter clinical trials, IPF continues to exercise a heavy human, financial and societal toll on its victims, their loved ones and their communities in which they work and live. With a gradually increasing worldwide [6] incidence and in view of the current disappointing status of available pharmaceutical agents the need for developing new treatments for IPF that are safe, effective and tolerable is now more challenging than ever [7].

Regenerative medicine and particular adult stem cells represent one such alternative option with several fruitful therapeutic applications in patients suffering from chronic lung diseases including IPF [8-19]. The past 5 years investigations of the therapeutic potential of adult stem cells and particular mesenchymal stem cells (MSCs) in experimental models of chronic lung diseases have expanded rapidly [15]. MSCs are stromal cells that can be readily harvested from numerous tissues, including bone marrow (BM), stromal vascular fraction (SVF) of the adipose tissue and cord blood [8,12,15]. Based on the recently published statement of International Federation for Adipose Therapeutics and Science (IFATS) and the International Society for Cellular Therapy (ISCT) [20], SVF is a supportive stroma lying within adipose tissue, which represents an abundant and easily accessible source of a heterogeneous cell population including hematopoietic and endothelial precursors as well as erythrocytes, fibroblasts, lymphocytes, monocyte/macrophages and pericytes [20-27]. However, the most important cell subpopulation of SVF is adipose-derived stromal cells (ADSCs) that seem to represent approximately 20% of initially isolated SVF cells. When SVF cells are seeded into culture they can be further purified from hematopoietic cellular components allowing increased expression of stromal markers (CD29, 73, 13, 90, 105 greater than 80% of cells) and progressive loss of stem cell associated (CD34) and hematopoietic (CD45) markers, a phenotypic profile resembling that of BM-MSCs.

Based on their unique pleiotropic paracrine properties [28-32], BM or umbilical cord MSCs, and adipose derived stromal cells - stromal vascular fraction (ADSCs-SVF) have been demonstrated to exert beneficial therapeutic effects in the experimental model of lung fibrosis [33-37] and emphysema [29,38,39], respectively.

On the contrary with pulmonary and critical care medicine, the use of stem cell therapy is now being established to patients suffering from complications following acute myocardial infarction [40-45]. Although efficacy results arising from these studies seem rather confusing and conflicting, all of them were characterized by encouraging safety data. The latter studies offered pivotal clinical insights and accidentally in one of them authors came up with an exploratory finding as they reported lung function improvement in the majority of patients [40]. This observation captured interest of chest physicians and triggered the launch of two clinical trials assessing the safety and efficacy of intravenous infusion of allogeneic BM-MSCs in patients with moderate and severe COPD. Encouragingly both studies reported an acceptable safety profile while none of them finally met efficacy objectives [46,47]. However, there is significant lack of knowledge regarding the exact fate of these cells within a fibrotic microenvironment, evidence that has limited their widespread clinical applicability.

To provide useful clinical insights that will help us to overcome major safety and ethical concerns accelerating the application status of stem cell therapy in IPF, we conducted a phase Ib non randomized, no placebo controlled, clinical trial to primarily study the safety profile of the endobronchial infusion of ADSCs-SVF, in patients with IPF of mild to moderate disease severity as assessed by functional status. Parameters related to functional profile, including forced vital capacity (FVC), diffusion lung capacity for carbon dioxide (DL_CO_), exercise capacity (6-minute walking test-MWT) and quality of life (Saint George’s Research Questionnaire-SGRQ were investigated as secondary exploratory end-points. Some of the results of these studies have been previously reported in the form of an abstract [48].

### Patients and methods

#### Trial design

**Figure 1:**
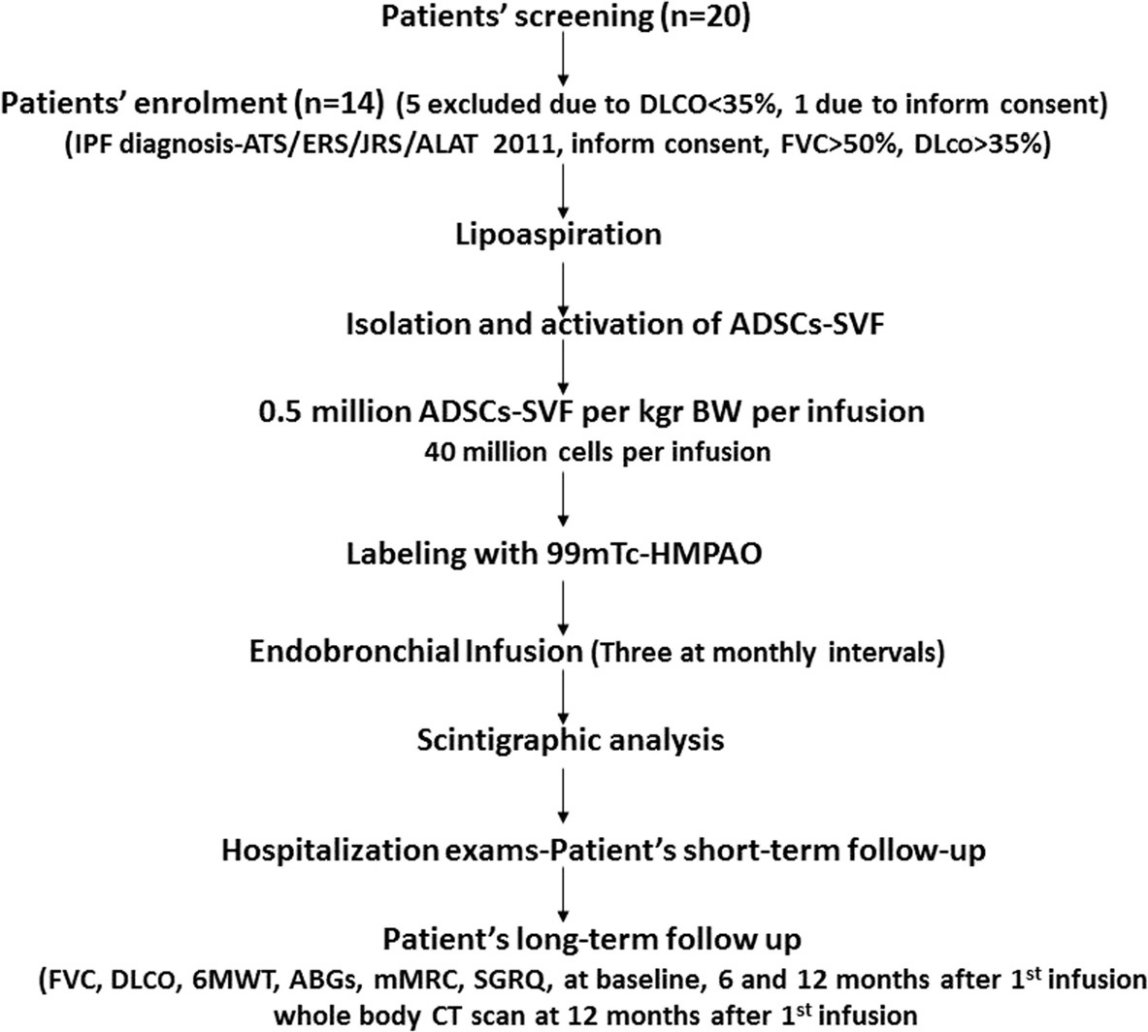
Schematic representation of the study protocol synopsis.

This study was a phase Ib, non-randomized, no placebo-controlled, unicentric clinical trial, conducted at the Department of Pneumonology, Medical School, Democritus University of Thrace and University Hospital of Alexandroupolis, Greece. Our study followed an already published protocol (inclusion, exclusion criteria, primary and secondary end-points) with slight modifications [13]. Our primary aim was to investigate the safety profile of autologous endobronchially administered autologous ADSCs-SVF in patients suffering from IPF based on the recently published diagnostic criteria of ATS/ERS (2011) [2], of mild to moderate disease severity as estimated by functional parameters including FVC >50% and DL_CO_ >35% of the predicted normal values. Our exploratory secondary goals were to assess efficacy of stem cell infusion based on functional, exercise capacity and quality of life criteria analyzed below. Between June of 2010 and September of 2011, a total of 20 patients were screened for enrolment in the study. Among them, 5 were excluded due to DL_CO_<35% and 1 refused to sign a consent form (Figure 1). Finally, 14 patients were enrolled in the study. The trial was conducted in compliance with current Good Clinical Practice standards and in accordance with the principles set forth under the Declaration of Helsinki (1989). The study was approved by Local Ethics Committee and the institutional review board of the University Hospital of Alexandroupolis, Democritus University of Thrace (EHD33/1SC/16-02-2010) before the initiation of patient enrollment. All patients entering the trial agreed to and signed an institutional review board approved statement of informed consent.

#### Lipoaspiration, isolation, activation, characterization and endobronchial infusion of ADSCs-SVF

**Figure 2:**
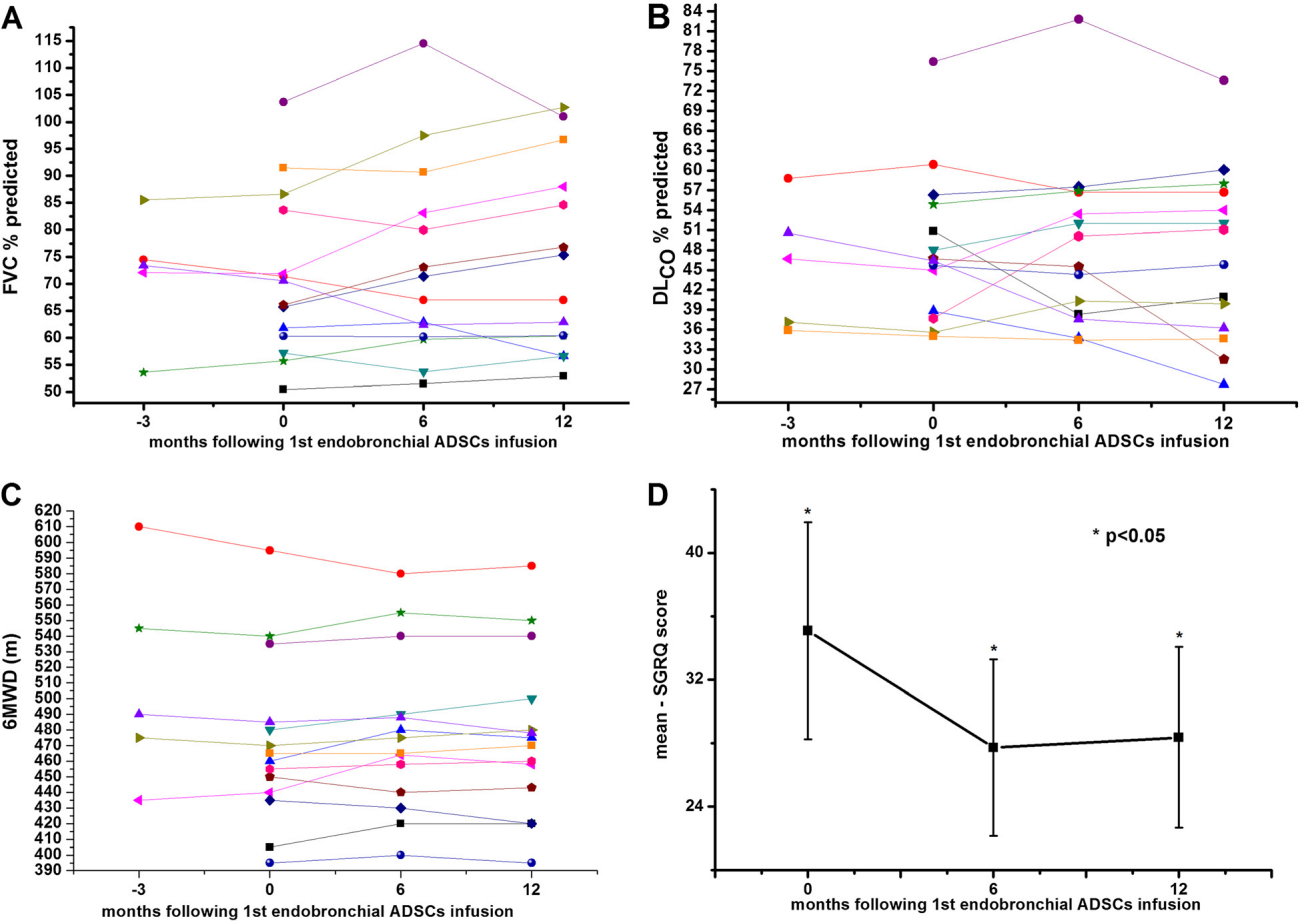
**Exploratory efficacy end-points before and after endobronchial infusion of adipose derived stromal cells-stromal vascular fraction (ADSCs-SVF). A**. Forced vital capacity (FVC)% pred. over time for each subject. Each line represents measurements made in a single subject. A time point 0 months indicates when first endobronchial infusion of the ADSCs-SVF was performed. As depicted, there were no statistically significant alterations between baseline and after 6 and 12 months following 1^st^ endobronchial infusion. A time point −3 months indicates period of time prior treatment initiation. **B***.* Diffusion lung capacity for carbon monoxide (DL_CO_)% pred. over time for each subject. Each line represents measurements made in a single subject. As depicted, there were no statistically significant alterations between baseline and after 6 and 12 months following 1^st^ endobronchial infusion. A time point 0 months indicates when first endobronchial infusion of ADSCs-SVF was performed. A time point −3 months indicates period of time prior treatment initiation. **C***.* 6-minute walking distance (6MWD) over time. As depicted, there were no statistically significant alterations between baseline and after 6 and 12 months following 1^st^ endobronchial infusion of the ADSCs-SVF. A time point −3 months indicates period of time prior treatment initiation. **D***.* Saint George’s Research Questionnaire (SGRQ) score over time. A time point 0 months indicates when first endobronchial infusion of the ADSCs-SVF was performed. As depicted, there was a statistically significant decline between baseline (0 months) and after 6 and 12 months following 1^st^ endobronchial infusion. *p<0.05.

ADSCs-SVF were obtained by lipoaspiration, isolated and activated according to an already published protocol with slight modifications [13]. In order to increase the therapeutic paracrine, anti-inflammatory and anti-apoptotic, potential of isolated cells, we applied a two-step activation procedure on the date of administration: a) Activation through autologous platelet rich protein (PRP) [49]. b) Activation using low level laser irradiation (5J/cm^2^) [50,51]. Following lipoaspiration, isolation and activation, a small volume of suspension (0.1cc) containing isolated cells was used for flow cytometry analysis on an epics Beckman Coulter flow cytometer. Since adipose derived-SVF consists of a heterogeneous cell population and to better define isolated cells we used a complete panel of differentiation markers comprising of the minimum required mesenchymal markers (CD29, CD73, CD90, CD105) [52] coupled with additional mesenchymal stromal cell markers including, CD44, CD13, CD116, as well as markers for leukocytic, hematopoietic and endothelial precursor markers, namely CD45, CD34 and CD31 (Figure 2). After isolation, activation and characterization our cellular sample was tested for pyrogenicity, using a commercial limulus amebocyte lysate (LAL) test (LAL chromogen end point assay Hycult Biotech Plymouth Meeting PA USA). Our final sample was diluted 1/10 in normal saline and then injected through the bronchoscopic channel in both patient’s lungs (final volume of 5cc in each lung). The activation, characterization and infusional procedures were performed thrice at monthly intervals. Details can be found in Additional file 1.

#### Labeling of ADSCs-SVF with ^99m^Tc-HMPAO (^99m^Tc-ceretec) and scintigraphic analysis

To visualize isolated cells within both lungs, in a representative number of patients (n=4), we labeled them with hexametazine ^99m^Tc-HMPAO (trade name Ceretec, a lyophilized molecule that helps ^99m^Tc to enter within the cell membranes) according to a modified protocol [53]. Retention of radiolabeled cells (99mTc-HMPAO) within both lungs was estimated with computerized image analysis by drawing regions of interest and calculating the average counts/pixels (average count). Details can be found in Additional file 1.

### Treatment group

Based on already published data showing an acceptable safety and efficacy profile of intravenously administered dose regimens of approximately 1.5 million BM-MSCs per body weight in patients suffering from either COPD [47] or myocardial infarction [41] we decided to administer in both patients’ lungs an overall of 1.5 × 10^6^ ADSCs-SVF per kgr of body weight divided into three doses, meaning 0.5 ×10^6^ ADSCs-SVF per kgr of body weight per infusion in each patient with IPF.

All eligible patients underwent bronchoscopy using a flexible bronchoscope, under local anesthesia (xylocaine). The flexible bronchoscope was guided into the lower lobes of both lungs and 1 aliquot containing ADSCs-SVF diluted into 10 cc of normal saline 0.9% was infused using a small catheter (2.0mm of diameter) through the bronchoscopic channel. Procedure was repeated thrice for every patient at monthly intervals.

#### Primary and secondary end-points

1) Primary safety assessments included monitoring and recording of all adverse events and serious adverse events. Arterial blood gases coupled with clinical (Medical Research Council-MRC dyspnea scale), electrocardiogram and monitoring of vital signs (temperature, oxygen saturation, respiratory and heart rate) were performed during the first 24 hours after each endobronchial infusion. The patient was then discharged 24 hours post-bronchoscopy given that he/she was afebrile and hemodynamically stable, with no signs of infection or any type of allergic reaction. Whole body computed tomography (CT) scan was performed in all patients to determine any ectopic tissue formation at the end of the follow-up period, meaning 12 months after the first stem cell infusion. Patients were subdivided into three categories depending on the level of toxicity. Details can be found in Additional file 1.

2) As exploratory secondary end-points we investigated whether stem cell infusion exerted any beneficial effects as assessed by clinical (modified Medical Research Council-mMRC dyspnea scales functional (FVC, DL_CO_), exercise capacity (6-minute walking test-MWT) and quality of life (Saint George’s Research Questionnaire-SGRQ) parameters, at baseline and at serial time points (6 and 12 months after the first endobronchial administration) [13].

### Statistical analysis

Statistical analysis was performed using the SPSS 17.0 and OriginPro8 software. Summaries of continuous measures were presented as the mean and SD. Safety and exploratory efficacy secondary end-points were observed for each patient against the baseline values. Comparisons of changes from baseline conditions were analyzed using the Student *t* test (independent sample t-test between treatment groups and 2-tailed, paired for pooled analysis) with the Bonferroni correction. Where multiple comparisons were performed, analysis of variance (ANOVA) with repeated measures was employed. Testing was performed at a 95% significance level. A p value <0.05 was considered as statistically significant.

## Results

### ADSCs-SVF differential cell count and viability

After lipoaspiration, isolation and activation using autologous PRP and photobiostimulation, isolated cells were analyzed for the expression of stem-cell specific surface antigens. To better characterize our heterogeneous cell population included within adipose tissue SVF a complete panel of markers was used. In line with previous reports [21,27,54], including flow cytometry analysis revealed that the majority of isolated cells were positive for mesenchymal markers including CD29: 79.1% (65.2 – 86.7), CD73: 67.7% (55.2 – 79.1), CD90: 59.3% (45.3 – 69.4), CD105: 50.1% (48.7 – 58.2), CD44: 90,3% (88.5 – 93.6), CD13: 54.2% (37.2 – 67.1) and CD116: 60.8% (49.4 – 64.1) (median values-range) while they were negative for CD45 and CD31. In addition, the remaining cells expressed CD34 marker: 28.8% (21.2 – 37.1) (median values-range), indicating the presence of hematopoietic and endothelial precursors. Based on the recently published statement of IFATS-ISCT [20] immunophenotypic profile of isolated cells (CD29+, CD73+, CD90+, CD105+, CD13+, CD44+, CD116+, CD34+ and CD31-CD45-) following their activation was consistent with the definition of adipose derived stromal cells lying within SVF (SVF subpopulation is additionally characterized by CD45 positivity) and therefore infused cells were defined as ADSCs-SVF. Cellular population exhibited an 80% consistency among patients enrolled, as shown in Table 1.

**Table 1:** Phenotypic characterization of ADSCs-SVF after isolation and activation with PRP and photobiostimulation in all patients enrolled (n=14) in the study

	**Differentiation markers**									
**Patients**	**CD29**	**CD13**	**CD73**	**CD90**	**CD105**	**CD116**	**CD44**	**CD34**	**CD31**	**CD45**
**1**	65.2	41.8	61.6	60.1	49.2	59.1	88.5	21.2	0.8	0.1
**2**	78.3	50.3	66.8	45.3	53.1	56.2	91.2	25.5	0.5	0.2
**3**	81.5	47.9	56.3	57.6	47.7	59.2	92.1	31.1	0.3	0
**4**	79.9	51.8	66.2	61.8	50.6	61.8	90.2	35.6	0	0.1
**5**	80.4	37.2	55.2	45.7	48.7	49.4	92.7	22.2	0.2	0.5
**6**	82.2	54.1	78.9	69.8	54.5	63.9	91.4	35.7	1	0
**7**	81.6	53.3	76.6	68.8	49.9	62.8	88.6	27.9	0.7	0
**8**	86.7	56.3	68.7	59.9	51.4	62.6	90.1	33.1	0.8	0.2
**9**	77.4	43.6	57.5	64.1	49.4	62.7	93.2	27.9	1.1	0.1
**10**	78.8	58.7	66.9	52.8	45.1	62.9	88.9	21.6	08	0.1
**11**	76.9	60.1	77.9	59.6	48.8	59.6	89.4	26.4	0.9	1
**12**	78.9	51.9	50.9	59.9	52.2	63.8	90.6	37.1	0.9	0.3
**13**	80.3	52.8	79.1	61.7	49.8	64.1	89.3	25.4	1	0.2
**14**	79.7	49.6	75.8	65.5	51.8	63.7	88.8	30.5	0.6	0
**Median % (range)**	79.1	54.2	67.7	59.3	50.1	60.8	90,3	28.8	0.7	0.2
	(65.2 – 86.7)	(37.2 – 67.1)	(55.2 – 79.1)	(45.3 – 69.4)	(48.7 – 58.2)	(49.4- 64.1)	(88.5 – 93.6)	(21.2 – 37.1)	(0–1.1)	(0.2-1)

Abbreviations: *ADSCs-SVF* Adipose derived stromal cells-stromal vascular fraction.

### Patients’ demographic data

All enrolled patients (n=14), completed all three endobronchial infusions. In addition, there was no patient that early terminated the study period, meaning 12 months follow-up period after the first endobronchial infusion. Demographic and baseline patient data are listed in Table 2. The majority of patients were males (12/14, 86%) while all patients were ex-smokers. All cases had a typical pattern of usual interstitial pneumonia (UIP) in chest HRCT scan. In 6 cases diagnosis of IPF was based on both radiological and histological criteria while 4 patients (28.5%) were characterized by the presence of upper lobe emphysema in chest HRCT. 5 patients were subjected to VATS in another department before visiting our department for further consideration. IPF diagnosis in this group of patients (based on radiological and histopathological criteria) was set before current guidelines were published. All patients were of mild to moderate disease severity based on functional data with baseline values in FVC%pred: 71.2±15.2 and DL_CO_%pred: 48.4±11.1. In addition, all patients were in good clinical condition walking approximately 475 meters during 6-minute walking test while none of the patients exhibited elevated levels of sPAP. Despite that, it is worth reporting that patients exhibited rather increased scores in indicators of quality of life, namely SGRQ (35.1±6.8). Five out of 14 patients (33%) were previously under low doses of corticosteroids (10 mg of prednisolone daily) and high-doses of N-acetylcysteine (1800 mgr per day) prior study enrolment and therefore entered a 6 week wash-out period of time before entering the study. The remaining 9 patients were treatment naïve by the time of study enrolment.

**Table 2:** Characteristics of the study population 3 months prior treatment initiation, at baseline and during follow-up period (6 and 12 months after first endobronchial infusion of ADSCs-SVF)

**Characteristics**	**3 months prior infusion**	**Baseline data**	**6 months post-1**^**st **^**infusion**	**12 months post 1**^**st **^**infusion**
Total number of patients	5	14	14	14
Male	4	12	NA	NA
Body weight (kgr)	79.6±11.2	79.6±11.2	77.1±8.6	76.2±9.4
Age (yrs)	NA	64.4±7	NA	NA
Ex smokers	5	14	NA	NA
Current smokers	0	0	NA	NA
Previous treatment with corticosteroids	5	5	NA	NA
Other treatment (NAC)	5	5	NA	NA
HRCT	5 (typical UIP pattern)	14 (typical UIP pattern)	NA	NA
VATS	2 (typical UIP pattern)	6 (2 typical and 4 probable UIP pattern)	NA	NA
Emphysema (HRCT)	2	4	NA	NA
sPAP (mmHg) (cardiac echo)	29.6 ±9	29.6 ±9	31.5±7	32.4±4
FVC%pred	71.8 ±11.5	71.2±15.2	73.4 ± 18.1	74.4 ± 17.5
DL_CO_%pred	45.82±9.5	48.4±11.1	48.9 ± 12.8	47.3 ± 12.9
6MWD	501±67.9	472.1±55.2	477.1 ± 50.3	476.4 ± 51.9
mMRC	2.0 ± 0.3	2.1 ± 0.6	1.7 ± 0.6	1.7 ± 0.4
SGRQ	NA	35.1±6.8	27.8 ± 5.6*	28.4 ± 5.7*

Values are expressed as mean ± SD unless otherwise indicated.

Abbreviations: *6MWD* 6-minute Walking Distance, *ADSCs-SVF* Adipose derived stromal cells-stromal vascular fraction, *DL*_*CO*_ Diffusion lung capacity for carbon monoxide, *FVC* Forced Vital Capacity, *HRCT* High resolution computed tomography, *IPF* Idiopathic pulmonary fibrosis, *mMRC* Modified medical research council, *NA* Non applicable, *NAC* N-Acetylcysteine, *SGRQ* Saint George’s Research Questionnaire, *sPAP* Systolic pulmonary artery pressure, *UIP* Usual Interstitial Pneumonia, *VATS* Video-Assisted thoracoscopic surgery.

*p<0.05 was considered as statistically significant.

### Safety outcomes

An acceptable safety profile of stem cells’ endobronchial infusions was reported in all patients enrolled in the study. All endobronchial infusions were well tolerated and no serious or clinically significant side effects were reported during the entire study period and over the 72 infusions (14 patients with 3 infusions each). As shown in Table 3, there were no side effects of minor or medium severity, including allergic reactions, liver or renal abnormalities, oxygen desaturations, cardiac abnormalities such as electrocardiogram or heart rate changes in 12 out of 14 patients (86%). There were only two patients (14%) that experienced worsening of cough and dyspnea accompanied by heart burn, oxygen desaturation (94% - 92% while breathing room air) and increase in heart rate (from 75bpm to 95bpm) shortly after the first endobronchial infusion (30 minutes) that were successfully managed with oxygen supply without any additional treatment. The above events did not reach clinical importance of disease acute exacerbation, since a non-statistically significant functional decline of 4.4% of FVC%pred. between baseline and 6 months after the first infusion (71.4 vs. 67) or 177ml in absolute values (2877 vs. 2700 ml) in the first patient and a decline of 8.2% of FVC%pred. (70.6 vs. 62.4) or 360 ml (2900 vs. 2540 ml) in absolute values in the second patient, were noted. Furthermore, 7 out of 14 patients (50%) complained of transient fever that lasted 24 hours after each endobronchial administration and was partially attributed to the bronchoscopic procedure [55]. However, the possibility that cell treatment could be responsible for this minor side effect cannot be excluded. Finally none of the patients experienced any ectopic tissue formation as reported by whole body CT scan that was performed at the end of the follow-up period, meaning 12 months after the first stem cell administration. Additionally available safety data 24 months following first infusion also revealed no ectopic tissue formation.

**Table 3:** Side-effects following endobronchial infusion of the adipose derived stromal cells-stromal vascular fraction (ADSCs-SVF) in patients with IPF (n=14)

**Side-effects**	**Number of patients (%)**
Fever	7 (50%)
Worsening of cough	2 (14%)
Worsening of dyspnea	2 (14%)
Oxygen desaturations	2 (14%)
Cardiac abnormalities	0
Allergic reactions	0
Infections	0
Liver abnormalities	0
Renal abnormalities	0
Acute exacerbation/Hospitalization	0
Deaths	0
Ectopic tissue formation (24 months follow-up)	0

Abbreviations: *IPF* Idiopathic pulmonary fibrosis.

### Efficacy outcomes

**Figure 3:**
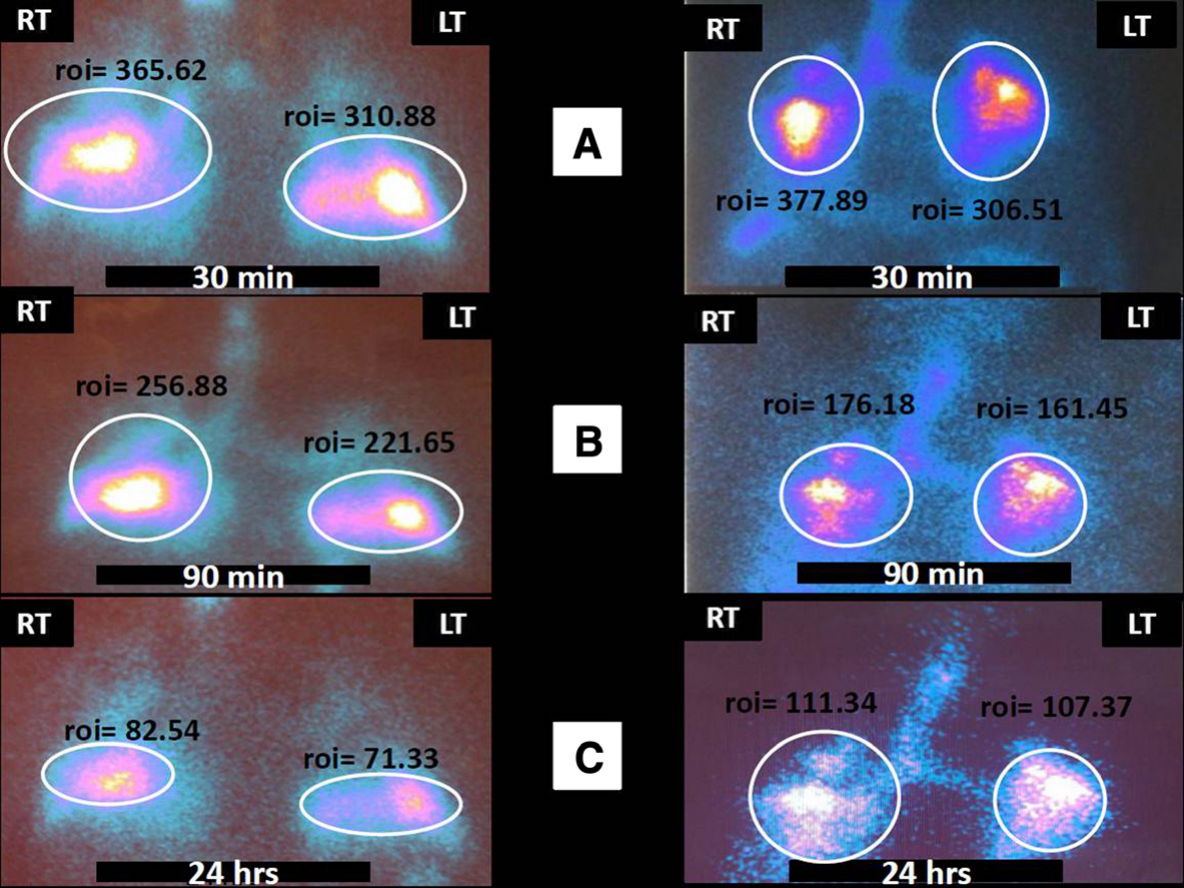
**99mTc lung scintigraphy at different time points (30 min, 90 min and 24 hours) after endobronchial infusion of the adipose derived stromal cells-stromal vascular fraction (ADSCs-SVF) in two representative subjects (right and left panel, A, B and C, respectively).** Retention of radiolabeled cells (99mTc-HMPAO) within both lungs was estimated with computerized image analysis by drawing regions of interest (roi) and calculating the average counts/pixels (average count). As depicted, signal intensity in both patients (right and left panel), although exhibited an expected decline through serial time-points (30 min vs. 90 min vs. 24 hours), nevertheless was present even 24 hours after the infusion. The latter evidence indicates the presence of ADSCs-SVF since free technetium (^99m^TcO_4_) has a half-time life equal to 6 hours and therefore it was impossible to produce signal 24 hours after the infusion.

There were no statistically significant differences in none of the studied functional parameters (FVC, FVC%pred. and DL_CO_%pred.) at baseline and 6 and 12 months following 3 endobronchial infusions of ADSCs-SVF (Table 2, Figures 2A-D). Additionally, available data 3 months prior stem cell administration in 5 subjects that were finally enrolled in the study also revealed no functional deterioration (Figures 3A, B). In addition, no significant alterations in mMRC dyspnea scale were also noted at both serial time points of treatment follow-up period as well as prior treatment initiation (Table 2). Furthermore, no statistically significant or clinically meaningful differences were also observed in 6MWD at 3 months prior treatment initiation, at baseline and at 6 and 12 months post-first endobronchial infusion (mean improvement of 5 and 4 m, respectively) (Figure 2C). Improvements in parameters of quality of life as estimated by a statistically significant decline in SGRQ (35.1±6.8 vs. p=27.2±5.6, p=0.02 and 35.1±6.8 vs. 28.4±5.7, p=0.02, respectively) scoring values, both after 6 and 12 months, were also noted (Figure 2D). Finally, a trend towards increase in systolic pulmonary artery pressure (sPAP) at 6 and 12 months post-first endobronchial infusion, although not statistically significant, was also noted.

### Scintigraphic analysis

As illustrated in Figure 3, 99mTc lung scintigraphy (anterior view) at different time points (30 min, 90 min and 24 hours) after endobronchial infusion of ADSCs-SVF in representative number of patients enrolled (n=4) demonstrated the presence of endobronchially infused radiolabeled ADSCs-SVF within patients’ lungs even 24 hours after the infusion. In particular, retention of radiolabeled cells (99mTc-HMPAO) within both lungs in two representative subjects (right and left panel, A-C, respectively) was estimated with computerized image analysis by drawing regions of interest and calculating the average counts/pixels (average count). As depicted in Figure 3, signal intensity in both patients (right and left panel), although exhibited an expected decline through serial time-points (30 min vs. 90 min vs. 24 hours), nevertheless was present even 24 hours after the infusion. The latter evidence indicates the presence of ADSCs-SVF since free technetium (^99m^TcO_4_) has a half-time life equal to 6 hours and therefore it was impossible to produce signal 24 hours after the infusion.

## Discussion

This is the first study investigating the safety profile of stem cell therapy in patients with IPF. The clinical trial met its primary objective demonstrating an acceptable and tolerable safety profile of endobronchially administered autologous ADSCs-SVF, during the infusions and during short- and long-term follow-up. Although this study was not designed to delineate mechanisms of actions or authenticate efficacy, it provides indications that current therapeutic approach may be of some benefit. In particular, almost all studied participants (86%) followed-up for 12 months exhibited stable functional and exercise capacity status, evidence that needs to be further explored. Comparisons of available clinical and functional data in 5 subjects 3 months prior treatment initiation and during follow-up period also revealed no deterioration. Improvements in indicators of quality of life (SGRQ) were also reported indicating a potentially substantial psychological impact of cell-based therapies in patients with devastating chronic lung diseases with treatment, yet, ineffective.

The present results accelerate the rapidly expanded scientific knowledge and establish a rigid basis for future efficacy trials investigating the therapeutic use of cell-based therapies in patients with chronic lung diseases, including IPF overcoming fears, ethical issues and safety concerns. The latter mainly arise from the absence of a coordinated statement regarding the exact mechanisms of action and fate of MSCs, within an inflammatory, fibrotic and dysplastic microenvironment that severely hampered clinicians’ efforts to study stem cell therapeutic potential in adult injured lung [8,12,15]. This rather disappointing ascertainment currently dominates respiratory research field despite the fact that a continuing accumulation of data supports both safety and efficacy of stem cell therapy in animal models as assessed by attenuation of experimental lung fibrosis and inflammation potentially mediated through MSCs protective paracrine properties [15,34-37].

In view of the significant lack of knowledge regarding the applicability of stem cell therapy in patients with IPF coupled with the current disappointing survival data arising from large multicentre clinical trials and the absence of a standard of care, we conducted a phase Ib, non-randomized, no placebo-controlled trial to study primarily the safety of the endobronchial infusion of autologous ADSCs-SVF in patients with IPF of mild to moderate disease severity (FVC>50%, DL_CO_>35%). As secondary exploratory endpoints we decided to include efficacy issues focusing on clinical (mMRC dyspnea scale), functional (FVC, DL_CO_), exercise capacity (6MWD) and quality of life indicators (SGRQ and CAT).

The most important finding of our study was the demonstration of an acceptable safety profile of adipose derived stem cell therapy locally administered within injured IPF lungs. Our approach exhibited a number of advantages including the following:

1) Firstly we used minimally manipulated autologous ADSCs-SVF lying in abundance within adipose tissue, thus eliminating the need for culturing over days to obtain a therapeutically viable number. In addition, cells were easily obtained by lipoaspiration, a procedure that is less painful than harvest of bone marrow. A growing body of evidence currently supports the notion that therapies involving minimally manipulated MSCs can overcome the fear and concern of undesirable alterations of allogeneic MSCs during ex vivo cellular expansion, including immunogenicity, contaminations, tumor, ectopic tissue formation and organ toxicity resulting from undesirable engraftment in the microvasculature [15,16,34,56-59]. In our study, ADSCs-SVF, were neither cultured nor expanded and were directly infused within the patients’ lungs after implementing a two-step activation procedure using a cocktail of autologous growth factors (PRP) [50] and photobiostimulation [51], two novel approaches known to amplify paracrine beneficial effects of MSCs. Furthermore, recent preclinical and human studies have raised significant concerns with regards to possible dysfunctional migratory and paracrine properties of ADSCs-SVF derived from older individuals [60] and mice [61]. In particular, Uji et al. [61] recently reported that stromal cells derived from the adipose tissue of relatively older mice exhibited reduced migratory capacity and failed to attenuate bleomycin-induced lung fibrosis. While the subject of an ongoing study, cultures of ADSCs-SVF derived from representative patients with IPF revealed a significant increase in the number of ADSCs-SVF colonies and an overexpression of anti-inflammatory (IL1-receptor antagonist) and angiogenic (vascular endothelial growth factor-VEGF) mediators, following activation with PRP and laser irradiation. Furthermore, no induction in the expression of mesenchymal markers (transforming growth factor-TGF-b, a-smooth muscle actin) or phenotypic characteristics compatible with fibroblast differentiation were observed (data not shown). In line with extended experimental [29,34,36,37] and human data [40,42,43,47,56,57] our patients did not experience any serious or clinically meaningful side effects, both during short-and long- term follow-up period, since no infusional toxicities, allergic reactions, disease acute exacerbations or ectopic tissue formation and tumor development were observed to date (24 months after the first infusion).

2) Secondly, in order to better characterize our isolated cell population and given significant controversies regarding isolation and characterization procedures between different laboratories we applied the most recent statement of IFATS/ISCT [20] and used a complete panel of surface antigens (n=10). Based on the recent definition of IFATS/ISCT our isolated cells, after being activated, developed characteristics of an immunophenotypic profile that lies between crude SVF cells and ADSCs for the following reasons: a) Consistently undetectable levels of CD45+ cells (characteristic of initially isolated SVF cells), b) levels of stromal markers (CD13, 29, 73, 90, 105) higher than those described in initially isolated SVF cells that did not reach peak levels seen in ADSCs which represent a more homogeneous cell population. c) Expression levels of stem cell associated marker CD34 were consistently high; however they were neither at peak values as observed in crude SVF population nor absent as noted in ADSCs. It seems that activation procedures may account for the temporal loss of expression markers that are present in the crude SVF cellular mixture rendering activated cells to exhibit a surface protein profile tending to resemble that of MSCs. Therefore, we applied the term ADSCs-SVF that better characterizes our isolated and activated cell population.

3) Thirdly, given that IPF pathogenesis is mainly restricted to the lungs and experimental data reports that intravenous stem cell administration is characterized by minimal lung uptake [62], we decided to deliver cells endobronchially in order to achieve maximum accumulation into sites of ongoing injury and thus, maximize their therapeutic potential. A semi-invasive technique of administration was chosen, namely bronchoscopy that was proven both well tolerable and accurate as estimated by scintigraphic analysis demonstrating prolonged strong signal intensity exhibited by radiolabeled stem cells and sustained even 24 hours after the infusion. Whether local administration of ADSCs-SVF will be proven more efficacious than systemic delivery remains to be elucidated.

4) Finally and most importantly we deliberately selected patients with mild to moderate disease severity since our study was designed to provide safety data and therefore events of disease acute exacerbation or progression that are more likely to occur in patients with end-stage lung disease could have masked our results. In addition, current experimental data demonstrates maximum beneficial effects during the early inflammatory stages of modeled disease that disappear later when established fibrosis has developed [36,63]. Nevertheless, the last parallelism is arbitrary and should be treated cautiously. Future studies are warranted to support this notion.

Despite relative enthusiasm arising from our safety data, our trial is underpowered and exhibits a number of caveats that should be addressed cautiously before rigid conclusions can be drawn. This trial has not been designed neither to investigate efficacy nor to elucidate mechanisms of stem cells’ actions. Therefore exploratory efficacy data presented here should be interpreted cautiously and rigid conclusions cannot be drawn safely. At this point it is worth reporting that statistically significant improvements in indicators of quality of life that were observed in almost all our patients (86%) may reflect a placebo-effect and deserve additional verification. Furthermore, any statements that arise from functional and exercise capacity outcomes indicating disease stabilization would be too speculative and therefore should be avoided for the moment. It is also debatable whether a trend towards increase in sPAP at 6 and 12 months post-first endobronchial infusion could be attributed to therapeutic interventions or simply reflects an epiphenomenon due to unreliable methods of sPAP assessment such as cardiac echo. Additionally available efficacy data 24 months after first infusion were excluded from current analysis since all enrolled patients after completing the 12 month follow-up period were switched to pirfenidone treatment based on the recently published CAPACITY trials [3] and therefore it was impossible to attribute any functional alterations solely to cell-based therapy.

Furthermore the use of a heterogeneous mixture of cell population such as adipose tissue SVF raises significant methodological limitations since the exact contribution of each one of them could not be delineated based on our study design. To substantiate our findings and better define our isolated cell population, we utilized a complete panel of mesenchymal, hematopoietic and endothelial markers. As shown in Table 1 and in line with previous reports [21,27,54], the majority of our non-cultured, non-expanded ADSCs-SVF were of mesenchymal origin, meaning that they were positive for the minimally required markers such as CD29, 73, 90, 105 as well as for CD44, CD13 and CD116. In addition, ADSCs-SVF after activation did not express surface antigens CD31, 45, as expected. Finally, almost a third of our cells stained positive for CD34 indicating the presence of hematopoietic and endothelial progenitors. One alternative approach was to purify MSCs by subtracting CD45-, CD31- CD34- cells using immunomagnetic beads. Nevertheless, we decided to avoid manipulation of isolated cells since by infusing unpurified ADSCs-SVF we exploited the beneficial stemness of the entire cell population including MSCs, lymphocytes, endothelial progenitors and hematopoietic stem cells.

While our results indicate some reassurance regarding endobronchial infusion of stem cells in patients with IPF, significant work is sorely needed to understand complex stem cell properties as well as their behavior and fate within a fibrotic microenvironment. Currently, whether MSCs could differentiate into fibroblasts [64] given their common mesodermal origin and accelerate fibrotic cascade or even promote tumorigenesis on a longitudinal basis, is under debate. Alternatively, we may speculate that ADSCs-SVF may exert their beneficial effects through their unique paracrine activities (anti-inflammatory, anti-fibrotic, anti-apoptotic and immunomodulatory) and less by acting as cells with regenerative capacity. In line with this premise and given a potential association between global immune impairment [65-67] and fibrogenesis we may speculate that a proportion of ADSCs-SVF may act as T regulatory cells [68] restoring immune deregulation and attenuating inflammatory and fibrotic cascade. The above statement is currently only speculative and needs further exploration. Studies are underway to shed further light into stem cells’ behaviour and resolve mechanistic issues surrounding their use.

## Conclusion

Collectively, this study was conducted to assess the safety of endobronchial delivery of autologous ADSCs-SVF in patients with IPF. Importantly our study met its primary objective and indicated an acceptable safety profile both with regard to acute infusions and during long-term follow-up. Detailed safety monitoring through several time-points indicated that cell-treated patients did not deteriorate, as assessed by functional parameters and indicators of quality of life. Whether these results represent placebo effect and bystander epiphenomena of an already prescribed favourable clinical course and not true outcomes of a therapeutic intervention remains to be proven. Our findings provide a way towards future, carefully designed, efficacy trials investigating the therapeutic use of cell-based therapies in patients with chronic lung diseases, including IPF overcoming steep barriers such as ethical issues and safety concerns.

## Abbreviations

6MWD: 6-Minute walking distance; ADSCs: Adipose derived stem cells; ADMSCs: Adipose derived mesenchymal stem cells; BM: Bone Marrow; DLCO: Diffusing lung capacity for carbon monoxide; HRCT: High resolution computed tomography; FVC: Forced vital capacity; KGF: Keratinocyte growth factor; mMRC: modified medical research council; MSCs: Mesenchymal stem cells; IPF: Idiopathic pulmonary fibrosis; PRP: Platelet Rich Protein; SGRQ: Saint George’s research questionnaire; SDF: Stromal derived factor; sPAP: systolic Pulmonary Artery Pressure; SVF: Stromal vascular fraction; VEGF: Vascular endothelial growth factor.

## Competing interests

Dr Tzouvelekis is a recipient of an unrestricted grant provided by Hellenic Thoracic Society for the years 2009–2012. Dr Paspaliaris and Dr. Dardzinski are employees of Adistem Ltd. Dr Koliakos is Head of the Hellenic National research foundation stem cell bank Athens, Greece. All other authors have no conflict of interests related to the topic of this manuscript.

## Authors’ contributions

AT: Study conception and design, data acquisition and interpretation, article draft, revision, and final approval. GK: Data acquisition and interpretation, stem cells isolation and flow cytometry analysis, article draft and final approval. VP: Study conception and design, article revision, and final approval. PN: Clinical data acquisition and interpretation and article final approval, AO: Radiological data acquisition and interpretation and article final approval. AZ: Scintigraphic analysis and stem cells radiolabeling and article final approval. NB: Scintigraphic analysis and stem cells radiolabeling and article final approval. GK: Data interpretation, article revision and final approval. BD: Study design and article final approval. DK: Lipoaspiration and article final approval. AA: Clinical data acquisition and interpretation and article final approval, MF: Study design, article draft, revision, and final approval. DB: Study conception and design, data acquisition and interpretation, article draft, revision, and final approval. All authors read and approved the final manuscript.

## Supplementary Material

Additional file 1 Lipoaspiration, isolation, activation, characterization and endobronchial infusion of ADSCs-SVF. Click here for file
