# Supplementary material for: A prospective, non-randomized, no placebo-controlled, phase Ib clinical trial to study the safety of the adipose derived stromal cells-stromal vascular fraction in idiopathic pulmonary fibrosis
Source: J Transl Med. 2013 Jul 15;11:171. doi: 10.1186/1479-5876-11-171 (PMC3722100; doi:10.1186/1479-5876-11-171)
Supplement: Additional file 1 — Lipoaspiration, isolation, activation, characterization and endobronchial infusion of ADSCs-SVF. [file 1479-5876-11-171-S1.docx]

**On line supplemental methods**

*Lipoaspiration, isolation, activation, characterization and endobronchial infusion of ADSCs-SVF*

Briefly, we report: All eligible patients underwent lipoaspiration under general anesthesia in a sterile surgical operating room setting. 50 mL of blood in EDTA was taken to prepare platelet rich plasma (PRP). A mean of 185 gr of adipose tissue was isolated from each patient from the above procedure performed by a plastic surgeon. Enzyme dissolution procedure was performed by incubating adipose tissue for 1 hour at 37^o^C under constant shaking with type I 100 U/ml collagenase (Biochrome). Adipose tissue was then centrifuged twice at 300 g for 30 minutes in order to separate the stromal vascular fraction (SVF) (pellet) from adipocytes. The SVF pellet was resuspended in phosphate buffered saline (PBS) and washed twice. The final pellet was re-suspended at 10 % patient’s serum in PBS. 10mL of the final suspension was used for bacterial check with the BacT/Alert system. A mean total of 1 x 10^6^ cells per gram of adipose tissue were isolated. The final pellet was re-suspended at 10% patient’s serum in PBS. A volume of DMSO solution was gradually added in the remaining suspension so that the final volume contains 10% DMSO and 2% Haes-steril 200. The cells were stored in three cryovials, air-tightly sealed with cryoflex membrane cryo-preserved at a 1^o^C per minute rate up to - 80 and then placed in constant temperature in liquid nitrogen and were stored until use.

In order to increase the therapeutic, paracrine anti-inflammatory and anti-apoptotic, potential of the isolated crude SVF cells, we applied a two-step activation procedure on the date of administration: a) Activation through autologous PRP [38]. One cryovial with isolated adipose derived SVF cells was rapidly defrozen for 1 min at 40^o^C washed once with 10% PRP in PBS and resuspended in 1mL PRP, derived from 50 ml of peripheral blood drawn from each patient, according to a standardized protocol. Final suspension was washed again with PBS and was ready for the next activation procedure. PRP as a storage vehicle of growth factors is a new application of tissue engineering which was considered for the application of growth factors (vascular endothelial growth factor-VEGF, keratinocyte growth factor-KGF, stromal derived growth factor-SDF) that may potentially accelerate proliferation of stem cells, improve their chemotactic activity and facilitate their engraftment to the multilayer vessels in order to exert their beneficial properties

b) Activation using low level laser irradiation (5J/cm^2^). The syringe containing isolated ADSCs- SVF enriched with PRP solution was placed for 20 minutes in a laser device specifically manufactured for the purposes of this study by Adistem Ltd. Photobiostimulation has been proposed to exert several biological actions including upregulation of angiogenic (VEGF) and anti-inflammatory mediators (IL-1a receptor) as well as down-regulation of inflammatory cytokines including tumor necrosis factor(TNF)- alpha and reactive oxygen species.

Following lipoaspiration, isolation and activation, a small volume of suspension (0.1cc) containing ADSCs-SVF was used for flow cytometry analysis on an epics Beckman Coulter flow cytometer. Since adipose derived-SVF consists of a heterogeneous cell population and to better define isolated cells we used a complete panel of differentiation markers comprising of the minimum required mesenchymal markers (CD29, CD73, CD90, CD105) [41] coupled with additional mesenchymal stromal cell markers including, CD44, CD13, CD116, as well as markers for leukocytic, hematopoietic and endothelial precursor markers, namely CD45, CD34 and CD31. The cells were incubated for 20 min at the room temperature with C29- fluorescein (FITC) (BD Biosciences, Heidelberg, Germany), CD73- FITC (BD Biosciences), CD90- FITC (BD Biosciences), CD105- allophycocyanin (APC) (BD Biosciences), CD44- FITC (BD Biosciences), CD13-FITC (BD Biosciences), CD116- FITC (BD Biosciences),CD45-PE (BD Biosciences), CD34-FITC (Miltenyi Biotec), CD13-FITC (BD Biosciences). Mouse IgG2a k –FITC (BD Biosciences) and PE Mouse IgG2a k –PE (BD Biosciences) were used as isotype controls. Viability of the cells was estimated by tryphan blue.

*Labeling of* ADSCs-SVF *with ^99m^Tc-HMPAO (^99m^Tc-ceretec) and scintigraphic analysis*

To visualize ADSCs-SVF within both lungs, in a representative number of patients (n=4), we labeled them with hexametazine ^99m^Tc-HMPAO (trade name Ceretec, a lyophilized molecule that helps ^99m^Tc to enter within the cell membranes) according to a modified protocol. A fraction of ADSCs-SVF was diluted into two 5cc syringes each containing sodium chloride 0.9% and incubated with two vials containing 0.5 mg of HMPAO reconstituted with approximately 2.0 mCi of free technetium (^99m^TcO_4_^-^), each and prepared to be infused within the right and left lung. Planar imaging of the lungs in anterior and posterior position was performed 30min, 90min and 24hours after the administration. Imaging was implemented in a single-head gamma-camera (GE Millennium MPS, Milwaukee USA) equipped with a low energy high resolution (LEHR) parallel-hole collimator. In order to estimate the retention of the radiolabeled cells in the lung in comparison to free technetium we drew regions of interest (roi) over the right and left lung in the posterior images at 30, 90min and 24 hours.

*Primary and secondary end-points*

Patients were subdivided into three categories depending on the level of toxicity, defined as:

1. **Low level**, including minor side effects such as increased cough, low fever (less than 37.5^0^C, skin allergic reactions)
2. **Medium level**, including non-life threatening allergic reactions, infections that do not require hospitalization, elevation of liver enzymes or creatinine serum levels.
3. **High level**, including death and/or life threatening major adverse events such as disease acute exacerbations as previously defined, or ectopic tissue formation compatible with tumorigenesis at the end of follow-up period (12 months after the first infusion)
